# A Rare Case of Persistent Left Superior Vena Cava Coexisting With Valvulopathies and Complete Heart Block

**DOI:** 10.7759/cureus.47245

**Published:** 2023-10-18

**Authors:** Sushil Paudyal, Amit K Thakur, Ahmed Z Abdelkarim, Faizan A Zakir

**Affiliations:** 1 Department of Cardiology, Quaid-e-Azam Medical College, Bahawalpur, PAK; 2 Division of Oral and Maxillofacial Radiology, Ohio State University, Columbus, USA

**Keywords:** cardiac surgery, complete heart block, mitral regurgitation, aortic stenosis, plsvc

## Abstract

Persistent left superior vena cava (PLSVC) is a rare congenital vascular anomaly that is often detected incidentally during cardiovascular imaging or interventions. Coexisting PLSVC with mitral regurgitation (MR), aortic stenosis (AS), aortic regurgitation (AR), and complete heart block (CHB) are exceptionally rare and have not been reported in the literature to our knowledge. We present the case of a 50-year-old male with PLSVC coexisting with severe MR, mild AS/AR, and CHB who successfully underwent permanent pacemaker (PPM) implantation and mitral valve replacement. Comprehensive diagnostic evaluation and tailored management strategies are crucial for achieving significant improvement in the patient's symptoms. The presence of PLSVC adds complexity to diagnosis and management, necessitating multidisciplinary collaboration for optimal patient care.

## Introduction

Persistent left superior vena cava (PLSVC) is a rare congenital vascular anomaly that results from failure of regression of the left anterior cardinal vein during embryonic development [1,2]. It is the most common thoracic venous anomaly, with an approximate incidence of 0.3% in the general population and up to 10% in patients with congenital heart conditions [1-3]. PLSVC usually drains into the right atrium via the coronary sinus (CS), but some studies show it may drain into the left atrium, creating a right-to-left shunt and cyanosis [1,2]. PLSVC is often asymptomatic and detected incidentally during cardiovascular imaging or interventions, such as pacemaker implantation, central venous catheterization, or cardiac surgery [1-3].

PLSVC can be associated with other cardiovascular anomalies such as atrial septal defect, bicuspid aortic valve, coarctation of the aorta, CS ostial atresia, and cor triatriatum [1,3]. However, to the best of our knowledge, the coexistence of PLSVC with mitral regurgitation (MR), aortic stenosis (AS), aortic regurgitation (AR), and complete heart block (CHB) is extremely rare and has not been reported in the literature. MR, AS, and AR are valvular heart diseases that frequently coexist and can lead to adverse cardiac outcomes such as left ventricular hypertrophy, heart failure, and sudden cardiac death [4,5]. CHB is a serious arrhythmia that necessitates permanent pacemaker (PPM) implantation for symptom relief and survival [6]. The presence of PLSVC can complicate the diagnosis and management of these conditions, especially during cardiac catheterization or surgery [6].

Here, we present the case of a 50-year-old male patient with PLSVC coexisting with severe MR, mild AS/AR, and previous CHB who underwent successful PPM implantation and mitral valve replacement. We also review the anatomy, diagnosis, and clinical implications of PLSVC and discuss surgical considerations for patients with this anomaly.

## Case presentation

A 50-year-old male presented to the Department of Cardiology on follow-up through OPD with complaints of shortness of breath, dizziness, and vertigo. The patient, devoid of any previous history of hypertension or hyperlipidemia, had stopped smoking eight years ago. He presented with a previous diagnosis of MR and CHB two months ago, and it was planned for PPM implantation. There was no significant family history of cardiac disease. Physical examination revealed a high-pitched, holosystolic murmur that was best heard at the left fifth midclavicular line and a prominent jugular venous pulse. The patient's vital signs were within normal limits. No other significant abnormalities were noted during the physical examination.

**Figure 1:**
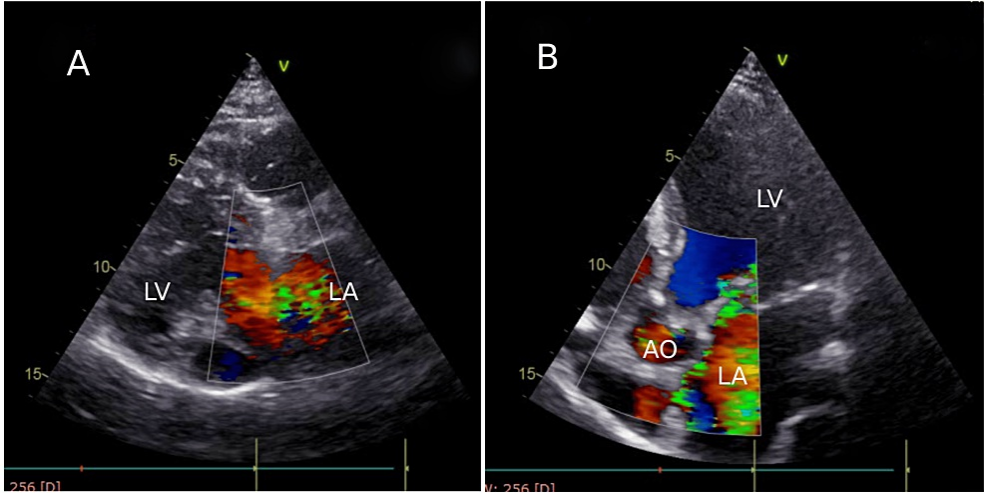
Transthoracic echocardiogram with color Doppler imaging A: Parasternal long-axis view with color Doppler showing a rapid jet of blood backflow in the LA, suggesting severe MR. B: Apical five-chamber view with color Doppler showing rapid, turbulent blood backflow from the LV into the LA, confirming severe MR. LV: left ventricle, LA: left atrium, AO: aorta

Echocardiography revealed a dilated left atrium (LA), dilated left ventricle (LV) with borderline LV systolic function, thickened and sclerosed mitral valve leaflets, and prolapse of posterior mitral leaflets into LA with suspicion of ruptured chordae with severe eccentric MR (Figure 1).

**Figure 2:**
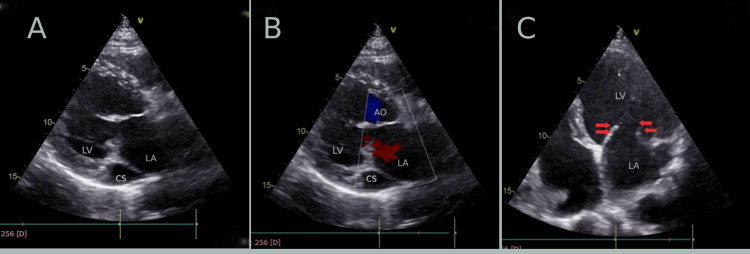
Transthoracic echocardiogram in various planes A: Parasternal long-axis view illustrating a dilated CS. Other chambers labeled are the LV and LA. B: Parasternal long axis view with color Doppler visible dilated CS and backflow of blood into LA on systole. C: Apical view which shows weak mitral valve leaflets (red arrows) with other structures visible: LV and LA. CS: coronary sinus, LV: left ventricle, LA: left atrium, AO: aorta

The CS was dilated, and there were thickened and sclerosed aortic valve leaflets with mild AS with a mean pressure gradient of 10.35 mmHg and mild AR. There was mild tricuspid regurgitation and moderate to severe pulmonary hypertension with a pulmonary artery systolic pressure of 59 mmHg. This echocardiography finding comes with the conclusion of significant mitral valve prolapse with severe MR, moderate to severe pulmonary hypertension, dilated right-sided chambers, dilated CS, and dilated LV with borderline LV systolic function. A dilated CS was a key factor suggesting the presence of an anomalous venous connection (Figure 2).

Further evaluation was performed using X-ray angiography, and a central venous catheter pass confirmed the coexistence of a PLSVC draining into the CS. The PLSVC originates from the left brachiocephalic vein and courses posteriorly to join the CS. The right superior vena cava was absent (Video 1).

**Video 1:** X-ray angiogram of the chest Chest X-ray angiogram demonstrated an unusual course of the central venous catheter with its distal end in the CS. Note the course of the catheter into the heart on the left side of the spine rather than on the right side.

Considering the symptomatic status, the patient was placed on a PPM. An aseptic procedure for the placement of a single-chamber PPM, a local anesthetic, was performed below the left infraclavicular region. A needle was then inserted into the left subclavian vein, followed by the passage of the guidewire and subsequent removal of the needle. An incision was made in the left infraclavicular region to create a pocket for pulse generation. The lead introducer was advanced into the left subclavian vein over the guidewire, after which it was withdrawn. The lead was carefully guided into the right atrium and adjusted to the apex of the right ventricle. The parameters were checked using a programmer to ensure their proper functioning. The lead was secured with sutures, and the pulse generator was connected. It was placed in the pocket, and the temporary pacing wire was removed with the aid of fluoroscopy. The incision was closed in layers, and the wound was dressed with an antiseptic covering. A pressure bandage was used to complete the procedure. The patient's postoperative course was uneventful. Regular monitoring and appropriate postoperative care were also provided. The patient experienced a significant improvement in symptoms, with a resolution of vertigo and dizziness. A mechanical prosthetic mitral valve was implanted through a median sternotomy. Follow-up echocardiography confirmed satisfactory functioning of the mechanical mitral valve with the support of PPM.

## Discussion

This case report presents a unique combination of cardiac anomalies in a middle-aged patient, including severe MR, AS, AR, CHB, and PLSVC opening into the CS. This combination of conditions is rarely observed in clinical practice, implicating the importance of comprehensive diagnostic evaluation and management strategies. MR is a serious valvular condition where blood backflows toward the LA, causing significant hemodynamic instability. AS, another common valvular heart disease, is characterized by sclerosis of the aortic valve that can lead to systemic hemodynamic dysfunction. In this case, the patient's AS was mild, with an aortic valve mean gradient of 10.35 mmHg and a calculated valve area indicative of significant stenosis coexisting with mild AR. This combination can exacerbate the hemodynamic burden on the heart. The presence of a CHB adds another layer of complexity as this can lead to significant bradycardia and associated symptoms, as observed in this patient's presentation with vertigo and dizziness.

Limited case reports in the literature describe PLSVC anomaly. Favale et al. [7] documented transvenous cardioverter defibrillator implantation in two patients with PLSVC and right superior vena cava (SVC) atresia. Al-Saloos [8] reported multiple congenital anomalies in a newborn baby girl, including levocardia, abdominal situs inversus, interrupted inferior vena cava, bilateral superior vena cava, right ventricular dilatation, and patent ductus arteriosus, with progressive right ventricular dilatation and spontaneous closure of patent ductus arteriosus observed during serial echocardiography. Li et al. [9] reported a case with PLSVC associated with anomalous right superior vena cava drainage, atrial septal defect, and atrial fibrillation, highlighting the importance of surgical intervention due to the risk of hemodynamic complications and atrial arrhythmias in such cases. Ichikawa et al. [10] noted the scarcity of studies reporting SVC anomalies, especially using multidetector-row computed tomography, in patients with horseshoe kidneys, although it is not directly relevant to our study, which indicates the novelty of our focus on this cardiac condition. Ari et al. [11] focused on determining the presence of concomitant PLSVC in patients with congenital heart disease. In an unrelated case, Ricciardi et al. [12] presented the management of a 33-year-old female undergoing hemodialysis with complete exhaustion of the brachial routes for vascular access who underwent long-term central venous catheter implantation using the echoscopic technique. Although not directly relevant, this study highlights the significance of vascular access in clinical situations. Other relevant studies by Garweg et al. [13], Povoski and Khabiri [14], and Davis et al. [15] contributed to a broader understanding of our case report.

This is the first case in which we demonstrated that PLSVC with coexisting MR, AS, AR, and CHB requires careful preoperative evaluation and planning. The choice of surgical or transcatheter intervention depends on the patient’s anatomy, hemodynamics, comorbidities, and preferences. The presence of a PLSVC may complicate vascular access and device placement in these procedures [16]. Therefore, multidisciplinary collaboration among cardiologists, radiologists, surgeons, and anesthesiologists is essential for optimal management of these patients.

## Conclusions

This case report highlights a unique combination of cardiac anomalies in an elderly patient, including PLSVC with MR, AS, AR, and CHB. The rarity of this coexistence draws attention to the importance of thorough diagnostic evaluation and tailored treatment approaches. The successful outcome of PPM implantation and mitral valve replacement indicates the importance of early detection and appropriate management in patients with such complex cardiac conditions. Multidisciplinary collaboration among cardiologists, radiologists, surgeons, and anesthesiologists is essential for optimal patient care, considering the complexity of the PLSVC and its potential impact on vascular access and device placement during surgical or transcatheter interventions. Further research and case studies will contribute to a broader understanding of this rare combination of cardiovascular anomalies and guide best clinical practices for similar patients in the future.
